# Health as a “global public good”: creating a market for pandemic risk

**DOI:** 10.1136/bmj.j3397

**Published:** 2017-08-31

**Authors:** Felix Stein, Devi Sridhar

**Affiliations:** Medical School, Edinburgh University, Edinburgh, UK

## Abstract

In the final article of the series **Felix Stein** and **Devi Sridhar** examine how the World Bank is trying to provide finance to improve preparedness for global pandemics

After the 2014 Ebola outbreak, the World Bank committed to providing a financial mechanism to support global pandemic preparedness. In line with its mandate of creating new markets, the bank is proposing an insurance arrangement that does not simply pool donor money but creates a market for private sector investment. We outline the bank’s efforts to do so through the Pandemic Emergency Financing Facility (PEF). We then analyse some potential benefits and wider concerns about private sector involvement in global health.

## Pandemic preparedness as a global public good

In reaction to the 2014 Ebola outbreak, a series of expert panels and committees recommended that a faster and larger international financial response to outbreaks should be part of pandemic risk mitigation.^1^ ^2^ The World Bank offered to provide a financial solution to support global pandemic preparedness and complement the work of the Global Health Security Agenda, which focuses on assessing domestic capacity in surveillance and support of health systems, and the World Health Organization’s health emergencies programme.

However, the World Bank’s involvement is more than an act of charity. It considers pandemic preparedness to be a “global public good.”^3^ ^4^ ^5^ ^6^ The theoretical framework around global public goods describes any material or immaterial entity according to whether it is excludable (ie, can a party be stopped from consuming it?) or rivalrous (ie, does its consumption reduce its availability for others or not?).^7^ ^8^ ^9^ Depending on these two features, economists often divide entities into four kinds—namely, private goods (eg, pills and syringes), club goods (eg, knowledge protected by patent), common goods (eg, universal healthcare), and public goods (eg, public information or pandemic preparedness) (fig 1).^10^

**Figure f1:**
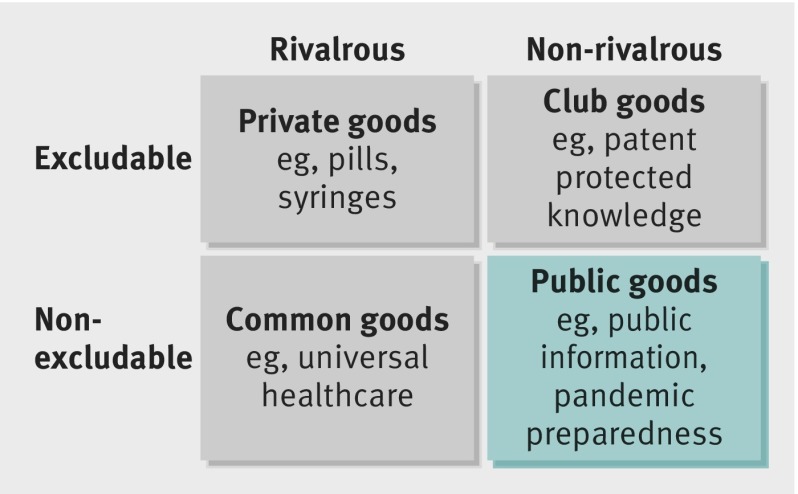
**Fig 1** Categorisation of global public goods.^7^ ^8^ ^9^ ^10^ Pure public goods are usually entities that are non-rivalrous and non-excludable

The bank’s description of objects and activities as “goods” reflects its economic approach to human health, in which market demand and supply affect healthcare provision. This approach was already laid out in 1993, when the bank gave its reasons for becoming involved in health, describing control of infectious disease as a highly effective, yet low cost, “investment” target.^11^ Seeing health as a driver of economic wellbeing also justified its major financial contributions during the fight against Ebola, which amounted to $200m (£150m; €175m) in August 2014, and over $1.6bn by mid-April 2015.^12^

## Creating a market for pandemic risk

In May 2016, the bank announced the creation of the PEF, a health insurance scheme for the world’s poorest countries and for qualified international responding agencies.^13^ ^14^ Recently introduced, the PEF should provide insurance coverage of initially up to $500m to countries eligible for support from the International Development Association (ie, the poorest countries that the bank works with) as well as United Nations agencies and other yet to be specified development and humanitarian aid organisations.^15^ This coverage may seem low, both because it is only 0.8% of the bank’s 2016 financial commitments worth $64.2bn and because the bank only pays for parts of it itself. Moreover, $500m is only a small fraction of the estimated trillions of dollars that a major influenza outbreak might cost.^15^

However, the PEF is supposed to act as an example because it sets out to to provide private sector funding for health in a new way. It draws on funds from reinsurance and bond markets to contribute to future costs of pandemics in the world’s poorest countries. Premiums are not paid by the recipients of risk coverage but by donors and private sector investors. The PEF is supposed to “scale up” response to an outbreak by attracting additional private funds for every donation it receives from the bank’s member countries.^15^ It is designed eventually to establish a new global market for pandemic insurance products, thereby increasing the coverage provided in the medium term.

The PEF consists essentially of two financial mechanisms, known as “windows” (fig 2). The first is the “insurance window,” which provides cover of up to $500m for infrequent, severe health pandemics. In an outbreak, part of the money it holds is paid out as long as the following criteria are met^15^: a country must be affected by a specific kind of pathogen, including orthomyxoviridae (eg, new influenza virus A, B, and C), coronaviridae (eg, severe acute respiratory syndrome, Middle East respiratory syndrome), filoviridae (eg, Ebola, Marburg), and other zoonotic diseases (eg, Crimean Congo, Rift Valley, and Lassa). Moreover, the size of an outbreak, measured in number of cases or of deaths, must be considerable (eg, 2000 confirmed cases worldwide for influenza), outbreak growth must be fast (eg, an increase of confirmed cases from 2000 to 5000 within a month), and spread of the outbreak must be broad (eg, two or more countries must be affected).

**Figure f2:**
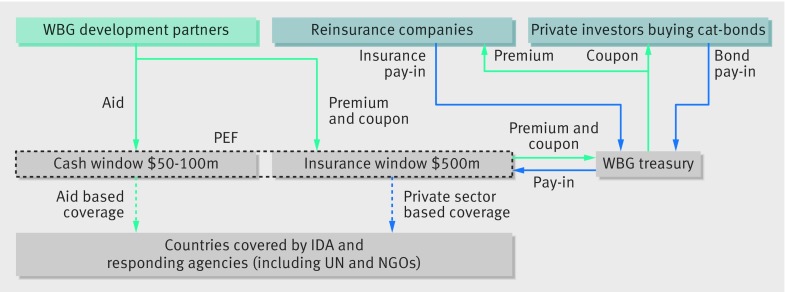
**Fig 2** Financial mechanisms of the PEF: “insurance window”, “cash window” (based on data from World Bank Group^15^). IDA=International Development Association; NGOs=non-governmental organisations; PEF=Pandemic Energy Financing Facility; WBG=World Bank Group; UN=United Nations

The second financial resource is the smaller and more flexible “cash window”, which provides insurance coverage of $50m-100m. This resource is financed entirely by donors, with no private sector involvement. It can be used when the payout criteria of the insurance window have not yet been met. For example, it provides money for severe single country outbreaks and for new or unknown pathogens. Moreover, in crises money can be paid out earlier and maybe even faster than from the insurance window, and can supplement financing for pathogens covered by the latter.^15^

## Potential benefits of the PEF

For donors, a big appeal of the PEF’s insurance window is that the bank’s development partners pay only the interest needed to attract private investment. In return, reinsurance companies and investors on capital markets provide them with a multiple of their payments in risk coverage. Moreover, the PEF promises to greatly “increase the speed of payment”—as this parametric insurance can be settled within days of activation criteria being met, instead of waiting until damages have been identified. It may even enable an “objective benchmarking of risk”—since payout criteria are not explicitly linked to the economic or political situation of covered countries.^15^ Finally, it is meant to incentivise countries to improve surveillance and early warning systems, in line with the International Health Regulations.^16^

Investors on capital markets, who are always eager to diversify their investments, may also find it appealing. They will be given the option of buying “pandemic catastrophe bonds” issued by the bank treasury. If they do, and a health pandemic breaks out with the insurance window becoming operative, they lose part of the invested capital (the “principal”). Yet, if the insurance window is not paid out, they get the full principal back after three years. While investors hold on to the bond and risk losing their money, donors are meant to pay them an annual interest (a “coupon”) via the bank’s treasury. The financing from reinsurance companies is similar, in that it provides coverage based on the same payout criteria, in return for annual premiums.

The idea of raising private money to reduce risk of a pandemic may find widespread approval. People in developing countries, as well as their governments, are likely to appreciate free financial risk coverage, and donors will be happy that the bank can raise private sector funds for each dollar given to the PEF. The PEF is also meant to smooth the boom and bust cycles of donor willingness to give, and provide a health related service that may hitherto have been underfunded because it was invisible until an outbreak.^17^ Moreover, it is likely to strengthen the bank’s own position in the health development sector and allow it to live up to its mandate of fostering economic growth. If the bank decides to charge fees for its financial services, the PEF might even constitute a source of bank profit.

The private sector has also large business interests in epidemic preparedness, response, and recovery. It knows that epidemics are bad for business. A World Economic Forum/Boston Consulting Group report noted that they “can have a major impact on employees, customer bases, and operations more broadly. Epidemics can devastate economies and threaten major investments by multinationals and small businesses alike.”^18^ Moreover, the bank promises that apart from diversifying private investor portfolios, “investing in preparedness yields significant returns”.^15^ It estimates that at a targeted maximum coverage of $500m for three years, annual interest payments by donors to the private sector should be in the range of $55m-$65m (ie, a lucrative 11-12% of coverage).^15^

The bank has provided a hypothetical example for how the PEF would work during an Ebola outbreak. As soon as Guinea, Sierra Leone, and Liberia notify the WHO of more than a total of 250 cases—that is, an increase of over 90%—the bank’s PEF coordinator would get in touch with the private company AIR Worldwide, which would then verify and declare that a payout threshold has been reached. The following day, $140m could be paid out from the insurance and the cash window. If confirmed cases and deaths rise further, additional payouts would be triggered, according to the currently unspecified payout procedures, disbursing hundreds of millions of dollars among, in this instance, the affected countries, WHO, World Food Programme, and Unicef.^15^

## Concerns about the PEF

The PEF raises several broad concerns. First and foremost, donors worried about the next epidemic outbreak may prefer to focus on more concrete preventive measures rather than insurance. Risk mitigation does not have to be financial, but can come in the form of trained health workers, clinical capabilities, health surveillance systems and laboratory networks, to provide just a few examples.^19^ ^20^ ^21^ ^22^ ^23^ ^24^ Whether delays in responding to Ebola were primarily of a financial nature remains contested,^19^ ^20^ ^21^ ^22^ ^23^ ^24^ and putting money into a finance mechanism rather than using it for other forms of risk mitigation may not be appealing to donors.

Moreover, private sector involvement in pandemic insurance provision greatly complicates the already convoluted structures of the health development sector. Introducing private investors with their own set of interests and a penchant for privatising knowledge into an industry filled with multilateral development agencies, charities, non-governmental organisations, healthcare providers, pundits, and state officials does not promise greater transparency or efficiency.^25^ ^26^ Importantly, potential donors will need to assess whether the insurance premiums and bond coupons they are supposed to pay for risk coverage are justified. Since the likelihood of a pandemic is largely unknown, this assessment relies on risk calculations based on data and calculative logics first established in the bond and insurance market of natural catastrophes.^27^ Development partners might not want to engage in this business of “fat tail risk assessment” (ie, estimating the risks of extremely unlikely but very costly events), which has only recently developed for earthquakes and hurricanes, and continues to be based on insufficient data and evolving mathematical logic.^15^ ^27^ It may be hard for donors to ensure that investors and reinsurers do not overcharge them as the latter have been in the business of risk assessment for decades, dedicate entire research departments to risk pricing, and often collaborate closely with companies specialising in pandemic risk assessment.

Moreover, the PEF’s payout methods as well as underlying risk models have mostly been developed by a working group consisting of the bank, WHO, and three private sector firms—Munich Re, Swiss Re, and AIR Worldwide.^15^ Thus, insurance cover and payout thresholds have been established “based on the epidemiological characteristics of the diseases [as well as the] affordability and risk appetite of investors and reinsurers” with little to no public oversight.^15^ It is still unclear to what extent investor interests have been written into the PEF, a question that merits donor attention since Munich Re and Swiss Re are themselves potential investors. This question also raises concerns as to the degree to which WHO’s mandate as the global arbiter of health emergencies, through its power to declare a public health emergency of international concern, has been curtailed.

Even if bank donors had no information deficits in relation to the PEF, it remains unclear whether a “market” for pandemic insurance provision by the private sector even exists. Donor country governments like Germany and Japan tend to have a lower cost of capital than many private sector institutions and can generally obtain money on capital markets more cheaply than private borrowers. So should these governments really pay private investors to provide the money for them?

Several ripple effects and unintended consequences are also worth thinking about.^28^ A financial market for pandemic risk management may replace the boom and bust cycles of donor willingness to give with the willingness of finance professionals to invest.^29^ As part of this development, risk analysts and investors will incorporate the opinions of healthcare practitioners into their market assessments, turning public statements of health experts as well as WHO data into financial market indicators worth hundreds of millions of dollars.

Moreover, people in the world’s poorest countries who will be considered “covered” by the PEF once it is active might want to know how their coverage operates. This knowledge is currently not available to them, as more detailed information about the model that determines when and how the insurance is paid remains proprietary and is held by AIR Worldwide, a catastrophe risk analysis company with headquarters in Boston.^30^ They might also wonder about the “moral hazard” of this new insurance mechanism. In an outbreak, will their governments continue to have their wellbeing at heart, even if just a few more cases of a certain disease may promise them millions of dollars by pushing them over existing insurance payout criteria?

Finally, unintended consequences could arise if the PEF bonds were to be bought and sold on secondary markets where investors can take a view on whether the PEF bond prices reflect the risk of epidemic outbreaks or not. In that case, we might find that some investors are short selling them, in the belief that the likelihood of a pandemic outbreak is higher than bond holders estimate. In this case, at least some investors will have a direct economic interest in seeing a large health pandemic happen.

## Market based solution to a public health problem

In conclusion, in declaring pandemic preparedness a “global public good”, the World Bank becomes more central in ensuring global health security. As a financial actor it also tries to provide a financial solution to a global health problem. Yet, in putting particular emphasis on market based solutions to health concerns, the bank risks creating a financial mechanism that is inefficient and opaque. This points to the wider tensions between the immediate pursuit of profit and the goal of providing healthcare to the world’s poorest people. Healthcare practitioners, donors, and people seeking healthcare should examine on a case-by-case basis how business and financial interests may or may not align with the goal of improving public health.

**Key messages**

The World Bank’s interest in “global public goods”, such as pandemic preparedness, reflects its role as an international financial institution active in global health.
The bank’s forthcoming Pandemic Emergency Financing Facility (PEF) includes private sector finance in order to establish new insurance markets
In a pandemic outbreak, the PEF promises speedy, large scale payouts, according to predefined criteria
The structure of the PEF raises serious concerns, which include pulling donor money away from preventing outbreaks, complicating healthcare financing, and possibly overcharging donors for risk coverage

## Notes

Contributors and sources: FS collected the data, analysed it, and drafted the initial version of the paper. DS helped conceptualise and design the analysis and revised the draft. FS is an economic anthropologist studying the World Bank. His research focuses on the moral and financial aspects of global health economics. DS holds a Wellcome Trust Investigator Award on the role of the World Bank in global health and is the coauthor of *Governing Global Health: Who Runs the World and Why? *(OUP, 2017). Data analysed for this series included World Bank financial datasets, archival sources, publications and reports, and staff interviews.

Provenance and peer review: Commissioned; externally peer reviewed.
